# Reproductive management in dairy cows - the future

**DOI:** 10.1186/s13620-017-0112-y

**Published:** 2018-01-08

**Authors:** Mark A. Crowe, Miel Hostens, Geert Opsomer

**Affiliations:** 1 UCD School of Veterinary Medicine, University College Dublin, Belfield, Dublin 4 Ireland; 2 Faculty of Veterinary Medicine, University of Ghent, Salisburylaan 133, 9820 Merelbeke, Belgium

**Keywords:** Dairy cattle, Herd health management, Cow fertility, Reproduction, Breeding

## Abstract

**Background:**

Drivers of change in dairy herd health management include the significant increase in herd/farm size, quota removal (within Europe) and the increase in technologies to aid in dairy cow reproductive management.

**Main body:**

There are a number of key areas for improving fertility management these include: i) handling of substantial volumes of data, ii) genetic selection (including improved phenotypes for use in breeding programmes), iii) nutritional management (including transition cow management), iv) control of infectious disease, v) reproductive management (and automated systems to improve reproductive management), vi) ovulation / oestrous synchronisation, vii) rapid diagnostics of reproductive status, and viii) management of male fertility. This review covers the current status and future outlook of many of these key factors that contribute to dairy cow herd health and reproductive performance.

**Conclusions:**

In addition to improvements in genetic trends for fertility, numerous other future developments are likely in the near future. These include: i) development of new and novel fertility phenotypes that may be measurable in milk; ii) specific fertility genomic markers; iii) earlier and rapid pregnancy detection; iv) increased use of activity monitors; v) improved breeding protocols; vi) automated inline sensors for relevant phenotypes that become more affordable for farmers; and vii) capturing and mining multiple sources of “Big Data” available to dairy farmers. These should facilitate improved performance, health and fertility of dairy cows in the future.

## Background

Dairy herd health management is undergoing a period of radical change worldwide. The drivers of this change are many and include the massive increase in technologies to aid in dairy cow reproductive management, quota removal (within Europe) and the significant increase in herd / farm size. Following the removal of quotas in Europe many countries are expanding dairy production, for example Ireland has ambitious plans to expand dairy output by 50%, this is to be achieved by a combination of increased herd size and greater milk output per cow [1, 2]. The present paper aims to identify some of the changes that will facilitate increased output, enhanced dairy-cow-herd health and reproductive management.

Within Europe, dairy herd size and numbers have been largely static from 1984 until 2015. As from April 2015, quotas have been removed allowing the opportunity for expansion to occur, this is likely to take the form of both increases in cow yields and increases in cow numbers. In Ireland, the Food Harvest 2020 report [1] (and underpinned by the Foodwise 2025 report [2]) discusses the expansion of dairy output by 50% between 2015 and 2020. Similar expansion is occurring in the Netherlands, although total phosphate limits are posing a challenge to expansion of cow numbers in that country. This review will focus on developments in the areas of data management, nutritional strategies, genetic strategies, disease control, precision livestock farming (hormonal treatments and sensor technologies) and male fertility that will have potential impact on increased milk production, cow health and cow fertility.

### Genetic strategies to improve reproduction

Up to the early 2000s, dairy genetic selection programmes in dairy producing countries traditionally selected predominantly for milk yield often at the expense of other dairy relevant traits, including fertility and health [3–5]. Breeding programmes in the early part of this century started to include fertility (e.g., by including traits such as longevity and calving intervals) and health as part of the selection traits. Inclusion of these traits has served to reverse some of the earlier trends that gave rise to reduced fertility. Over the last 15 years it is now recognised that trends in both longevity (increased) and calving intervals (decreased) have improved [5]. A major challenge for breeding programmes in terms of incorporation of fertility traits has been to develop phenotypes that have reasonable heritability. For example many fertility traits have typically only low heritability estimates (e.g., 0.1, compared with many growth and carcass traits where the heritability is 0.25–0.5). A second major issue for many fertility traits is to have easily measured phenotypic traits or genomic markers (single-nucleotide polymorphisms; SNPs) that correlate to appropriate fertility traits.

Opportunities may now be arising for selection of new traits that could be incorporated into breeding programmes. An EU funded project “Genotype plus Environment” (GplusE) has amongst its many objectives the identification of new novel milk based phenotypes that may be used as predictors for the traditional, but as well as the difficult to measure, record and select traits such as conception rates and uterine health (www.gpluse.eu). This project aims to develop, amongst other things, novel milk based traits that correlate and predict health and fertility traits in dairy cows. The strategies being used include the measurement of Mid-infrared spectra (MIR) in milk, metabolites in milk and glycans on the immuno-gamma globulin (IgG) fraction of milk. The project is relating these new measurable traits to fertility and health traits [6–9], and then relating both novel and traditional traits to novel genomic markers (SNPs) eventually facilitating improved selection strategies in the future. This project and work from other labs should result in further fertility SNPs that may enhance genetic selection for additional improvements in fertility.

### New tools and applications to new phenotypes that may be used in the dairy sector

Recent work in University College Dublin has led to the development of glycan markers for uterine health. This has been developed into a patent application (PCT/EP2014/068734: “Methods for predicting, diagnosing or monitoring infections or conditions”). Indeed milk-based glycan markers have also been developed that can predictively identify cows having retained placental membranes [6]. Such biomarkers that are easily measured in milk would allow animal breeders to select for cows with a propensity for improved uterine health and therefore move towards cows that would have increased fertility.

While in-vitro fertilisation (IVF) and embryo transfer are now significant tools to increase genetic selection on the female side with *Bos indicus* cattle [10], currently multiple ovulation and embryo transfer remains the more cost-effective method for *Bos Taurus* cattle (including all significant dairy breeds: Holstein-Friesian, Brown Swiss, Jersey etc) at population level. This is because the *Bos Taurus* breeds only produce between 5 to 20 follicles per follicle wave emergence event [11], which is insufficient numbers to allow adequate numbers of ova for effective culture for IVF.

### Nutritional strategies to improve reproduction

Modern dairy cows have been predominantly selected for a high milk yield in early lactation that is associated with a very high capacity to mobilize body reserves during this period. In a study by Tamminga et al. [12] with 5 production trials using 295 cows, calculations showed that cows can produce as much as between 120 and 550 kg of milk from body reserves on the basis of energy (average 324 kg). Maximum mobilisation in 8 weeks amounted to 41.6 kg empty body weight, 30.9 kg fat and 4.6 kg protein [12]. Most cows can cope with this metabolic load which is defined as: ‘the total energy burden imposed by the synthesis and secretion of milk, which may be met by mobilisation of body reserves’ [13]. Metabolic stress however is defined as ‘the amount of metabolic load that cannot be sustained by this mobilisation, leading to the down-regulation of some energetic processes, including those that maintain general health’ [13]. Hence, the ‘over’ mobilisation of body reserves during the period of NEB is a key factor for disease susceptibility in modern dairy cattle. Furthermore, in addition to post calving energy balance, pre-calving loss in body condition also has significant consequences for metabolic status, milk composition and subsequent health [14] and should be acknowledged.

The genetically and hormonally driven body mobilisation is further aggravated by a serious mismatch between the energy need and the cow’s capacity to take in energy [15]. The latter often being even further negatively affected by an inadequate adaptation of both the gastro-intestinal tract and the overall intermediary metabolism and often an elevated incidence of diseases in the period after calving [15]. Maximal feed intake occurs commonly at 6 to 8 weeks in lactation, which is much later than peak production, causing cows typically to be in negative energy balance for 5–7 weeks post partum [12].

Components of reduced fertility in modern dairy cows include delayed resumption of normal ovarian cyclicity [16–18], uterine health [17–19], lower expression of heat symptoms and lower pregnancy rates to first and subsequent inseminations. The latter mainly being caused by an increased incidence of embryonic and foetal death [20]. Relevant review papers have been published about the mechanistic backgrounds of the relationship between metabolic stress and impaired fertility in modern postpartum dairy cows [21, 22].

Management strategies for transition cows are mainly focused on helping the cows to cope with the metabolic load by optimizing health, minimizing stress (e.g., by minimising the changes in group or ration), stimulating dry matter intake and immune function. There are great opportunities for the veterinary practitioner to regularly monitor and adapt the herd management in order to do so. LeBlanc [23] and Mulligan et al. [15] identified the key issues that should be covered by the practitioner to optimally guide farmer clients to optimize their transition-cow management.

Furthermore, application of diets specifically designed to improve fertility by counteracting mechanisms related to the negative energy balance (NEB) or by supporting a specific pathway that is necessary for successful fertility, has always been a very attractive way to circumvent the impairment of reproduction during early lactation [24]. Although the reproductive system is known to be influenced by multiple hormones that are also involved in the adaptation towards high milk production (e.g., growth hormone; GH, insulin-like growth factor I; IGF-I and leptin), only insulin is known to be relatively responsive to changes in the composition of the ration [25]. Ovarian follicles contain insulin receptors [26] and cows with lower peripheral insulin levels in the immediate postpartum period suffer from retarded postpartum ovarian resumption and normal cyclicity among others by a higher risk to suffer from cystic ovarian disease [27]. Therefore, glucogenic diets have been advocated in the immediate postpartum period aiming to enhance the peripheral insulin concentrations and advance normal ovarian resumption [25]. However, insulin has been shown to have detrimental effects on oocyte and embryo competence [28] and has been shown to stimulate enzymatic catabolism of progesterone (P4) in the liver [29]. The latter suggests glucogenic diets only being of advantage when offered in the immediate postpartum period, while they should be avoided when cows are inseminated.

Rations leading to high peripheral urea levels are generally mentioned to be associated with lower pregnancy rates due to its detrimental effects on the embryo [30]. However, the mechanistic pathways by which this detrimental effect may be caused and the threshold peripheral urea concentrations, are both still matters of debate. Special attention in this respect should be given to the supplementation of soyabean meal as the main protein source in the ration. In a recent study it was demonstrated that commercially available soyabean meal contains isoflavones in concentrations that are able to induce increases in the blood concentration of oestrogenically active isoflavone metabolites (equol, O-desmethylangolensin, dihydrodaidzein) in high yielding dairy cows post partum, even when supplemented in relatively low amounts (1.72 kg per day on average) [26]. When compared with rapeseed meal, soya supplementation was furthermore associated with a decreased angio- and steroidogenesis at the level of the corpus luteum (CL) based on biopsy sampling at day 9 of the oestrous cycle [31]. However, it was not possible to demonstrate any effect on the peripheral progesterone concentration during the first 3 oestrous cycles after calving [31]. Therefore, although the results of that study suggest negative effects of soya feeding on CL function in recently calved dairy cows, the contribution of this effect on the peripheral progesterone concentration and consequently on overall fertility of supplemented cows warrants further research [31].

Adding fats is another strategy that has been extensively tested to reduce the impaired reproductive capacity of dairy cows. A study aiming to minimize the negative energy balance by decreasing the milk fat synthesis and hence limiting energy output via milk by supplementing the ration with exogenous fats, was not successful since cows simply produced more milk when reducing the NEB [32]. Omega-6 fatty acids are believed to have pro-inflammatory and thus prostaglandin F2alpha (PGF)-stimulating properties rendering them of extra value early post-partum, while omega-3 fatty acids can weaken this inflammatory potency, leading to a higher chance of survival of the embryo when supplemented during the periconceptual period [33]. Unfortunately, research results rarely provide a consensus in this topic. The consequences of these fat-feeding strategies on oocyte and embryo quality remain an intriguing issue for debate. Fat feeding may alter the microenvironment of the growing and maturing oocyte of the early and older embryo and thus may affect reproductive outcome [34]. Research has shown that dietary-induced hyperlipidaemic conditions can be harmful for embryo development and metabolism [35]. However, to date, research results remain somewhat conflicting most probably due to differences in fat sources used, in diet and duration of supplementation and in experimental set-up in general [35]. Furthermore, peripheral blood in lactating dairy cows will contain a mixture of fatty acids of dietary origin and from body-tissue breakdown, the latter being largely abundant in the immediate postpartum period and containing a high proportion of saturated fatty acids [34, 36]. Especially the latter have been shown to have a significantly detrimental effect on both the oocyte as well as embryo quality [34].

Adding extra vitamins and minerals to the diet has often been suggested as a “golden bullet” solution to reduce declines in cow fertility by various commercial interests, while requirements for optimal reproductive efficiency in modern dairy cattle deserve careful re-evaluation based on well-designed scientific research [37]. Usually farmers readily adopt these “proposed supplement solutions” since they don’t involve extra labour which is often their paramount constraint. Stating whether the amount of these compounds is sufficient in the ration is often very difficult for the practitioner since it is usually impossible to even estimate the content of these substances present in the basic roughage ration. In herds in which cows are given high quantities of concentrates to sustain peak yield in the immediate postpartum period, the risk of suffering from specific deficiencies is lower due to the fact that concentrates are usually highly supplemented with vitamins and minerals [37]. In terms of their effect on immune response and embryo quality, special attention should be given to vitamin E and selenium. The latter was supported by recent finding that in herds that were tocopherol deficient during the dry period, treatment with injectable vitamin E of 1000 IU each week for the last 3 weeks of gestation not only reduced the incidence of retained placenta and stillbirth but also significantly decreased pregnancy loss (20.5% vs. 12.5%; *P* < 0.01) [38].

### Controlling infectious diseases

Veterinarians managing fertility in dairy herds should regularly evaluate the herd health status for pathogens known to compromise reproductive efficiency. Infections with pathogens like *Leptospira hardjo*, bovine viral diarrhoea or herpes viruses are known to reduce conception rates, while infections with *Neospora caninum* and emerging viruses like the bluetongue virus may cause foetal losses and abortions. Bovine herpes virus 4 is reported to have a tropism for endometrial cells and therefore should be specifically monitored and controlled in herds suffering from uterine diseases, particularly where other risk factors are controlled or ruled out [39]. In addition to continuing careful monitoring and appropriate biosecurity plans inclusion of appropriate vaccination protocols may be required to prevent the introduction of new agents into the herd and to prevent spread within the herd [40].

Of special interest among bacterial diseases, is the minimisation of uterine disease. In cattle, bacterial contamination of the uterus is ubiquitous at parturition. However, this does not automatically imply the establishment of uterine disease and subsequent fertility problems. It is generally a suppression in uterine immune function in addition to pathogen presence that allows a shift in bacterial populations and establishment of disease in up to 20% of animals [19, 42]. Despite the fact that several papers have been published aiming to come to a general agreement about the definitions of postpartum uterine diseases based on mainly clinical symptoms [41, 42], there is still a lot of confusion about these definitions among practitioners. This confusion in definitions gives rise to a wide variety of preventative and curative treatment protocols being applied in the field, many of which are not scientifically proven to be efficacious. Recent literature underlines the high incidence of especially subclinical endometritis in high yielding herds [43]. Diagnosis of this impairment is based on intra-uterine sampling for cytology, which is not routinely done at the moment. Therefore, work by Pascottini et al. [44] reported the use of the cytotape that allows sampling early post partum and during insemination, and facilitates profiling of uterine cytology in repeat breeder cows. The generally accepted necessity to minimise the use of antibiotics in cows should be extended to treatment of uterine infections. It is important to determine the risk factors for the different uterine diseases, and design prevention and control programmes to reduce the incidence of disease.

### Use of precision livestock farming

#### Oestrous detection

Traditional approaches to reproductive management and use of artificial insemination have included either visual observation of oestrous behaviour, or the use of fixed time insemination protocols (e.g., OVSYNC [45]).

To achieve high submission rates to artificial insemination (AI), which are critical to achieve a 365-d calving interval in seasonal calving herds, requires an effective, practical means of identifying each cow in oestrus. Standing to be mounted is considered the main behavioral sign identifying an oestrous period and is used to determine the correct time to inseminate [46]. Both the physical activity and mounting activity induced by increased oestradiol production during the preovulatory follicular phase can be monitored in various ways. Heat detection rates (submission rate) vary from herd to herd with between 30 and 70% of cows exhibiting oestrous behaviour usually being detected in oestrus. With optimal visual observation of mounting activity for 20 min 5 times per day heat detection rates of 90 to 95% may be achieved [47] but is considered laborious and time consuming. With lower frequency of observation, lower rates of oestrous detection are achieved especially with higher yielding cows (e.g., only 70% of cows detected in oestrus with two or three observation periods of 30 min duration [48]).

Furthermore, in high-yielding Holstein-Friesian dairy cows, the percentage of cows that display standing to be mounted by other cows has decreased, leaving it more difficult to detect oestrus [49]. Roelofs et al. [49] found that only 58% of cows were observed in standing oestrus. This, in turn, decreases submission rate to AI and thereby contributes significantly to reduced reproductive efficiency [50].

Successful reproductive performance based on detection of oestrous behaviour requires the need to accurately detect oestrous onset in the majority of cows, and then inseminate 4 to 16 h later [51]. This led to the common practice of breeding cows according to the am-pm rule which requires that cows are observed for oestrus five-times per day, those commencing oestrus in the morning get inseminated that evening and those commencing oestrus after 12.00 noon are inseminated the next morning (onset of oestrus defined as the first observation period where the cow is observed to stand to be mounted by other herd mates or a teaser bull).

The approach of oestrous observation has served well for herds prepared to invest the time and effort into good and accurate oestrous detection. However, it requires a significant commitment of labour, good cow identification and personnel trained in detection of oestrus in cows.

#### Sensors for oestrous detection

Over the last 2 decades various systems for automation of oestrous detection have been developed to various degrees of success.

##### Pressure sensors

The characteristic oestrous behaviour of standing to be mounted can be monitored through the use of systems such as, scratch cards (e.g., Estrotect; Rockway Inc., Spring Valley, WI), colour ampoules (Kamar Products Inc., Zionsville, IN), vasectomized bulls fitted with a chin-ball marker, the use of tail-painting methods or the electronic device HeatWatch [47, 48, 52].

##### Activity monitors

**Fig. 1:**
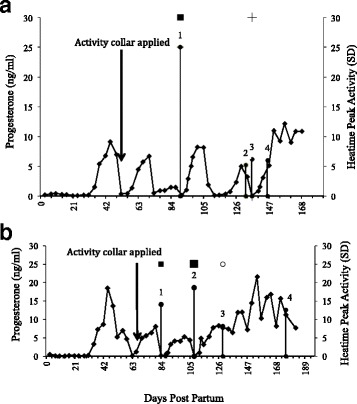
Milk progesterone profiles and activity clusters(*) associated with different reproductive states for two representative post partum dairy cows (**a** and **b**). Heatime™ activity clusters are labelled 1–4. Insemination with conception = symbol ■. Insemination and full term pregnancy resulting = symbol +. Insemination whilst pregnant and still went full term = symbol O. Aungier et al. [48]

**Fig. 2:**
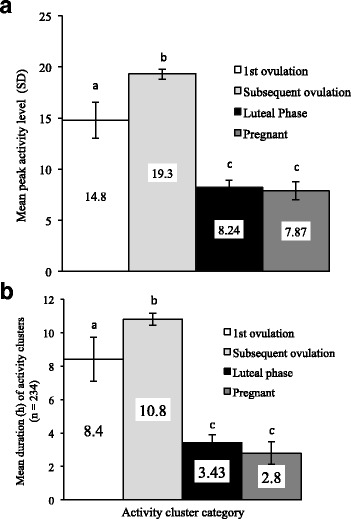
**a** The Mean ± SEM peak activity level of activity clusters was influenced by the endocrine state in which they occurred and **b** The Mean ± SEM duration of activity clusters was influenced by the endocrine state in which they occurred. ^a-c^Means within a bar chart with different superscripts differ (*P* < 0.0001). Aungier et al. [48]

One labour-saving technology available to farmers to help increase submission rate and decrease labour requirements for oestrous detection is the use of a monitor of physical activity. The pedometer, attached to a leg, detects an increase in the number of steps taken per hour during oestrus (e.g., S.A.E. Afikim, Kibbutz Afikim, Israel) [52], whereas the use of a neck collar (e.g., Alpro; DeLaval International AB, Tumba, Sweden; Heatime, SCR, Netanya, Israel; MooMonitor; Dairy Master, Ireland) [53] identifies increased physical activity (walking, mounting, getting up and lying down) expressed as an activity cluster (AC) and alerts the farmer as to when the AC started (when the cows next enter the milking parlour). It can therefore identify for the farmer the optimum time to AI, which is during a 12- to 18-h window before the predicted time of ovulation. A recent study, using the neck collar activity monitor Heatime (SCR Engineers Ltd., Netanya, Israel), identified that the odds of an AC being in a pre-ovulatory follicular phase rather than a luteal phase improved by 29% for every 1-unit increase in peak activity and by 91% for every 2-h increase in duration of an AC (Fig. 1, Fig. 2) [54]. Using one such activity monitor (Heatime) the optimum time to inseminate was between 9 and 15 h after the activity cluster was triggered [55].

##### Endocrine profiling

A commercially available in-line measurement system for endocrine profiling has been recently developed (Herd Navigator, Delaval) to detect metabolites and P4 concentration in milk [56]. Using algorithms P4 profiles may be used to predict oestrous events and potentially pregnancy status. However, to date this technology is still relatively expensive which is limiting its adoption. In addition, there are limits to its technical usefulness.

i) the system was initially developed assuming daily measurement of P4 in milk, however in the commercialised format it is often considered too expensive to use for daily measurements and generally only gets used twice or once weekly in herds equipped with this technology [56].

ii) The follicular phase in cattle can vary from 3 to 7 days and is highly variable, even with daily measurements the transition to the follicular phase (i.e., high P4 to low P4 is marked by the drop in P4) is not a good predictor of ovulation or onset of oestrus and is therefore not specific enough for timing of inseminations in practice. It can however identify follicular phase cows that should then be specifically observed for signs of oestrous behaviour (by other means) to then allow timing of insemination. Where measurement is only once or twice weekly this becomes much less useful and at weekly intervals the follicular phase can be inadvertently missed entirely.

iii) As a method for determining pregnancy status P4 is more reliable as a non pregnancy test than confirming pregnant positive cows. This is because a drop in P4 18–24 days after a correctly times insemination means non-pregnant. However high P4 18–24 days after an insemination can be due to pregnancy; or miss-timed initial insemination (meaning the cow is now in a non-pregnant luteal phase); or a persistent CL that appears as an early pregnancy profile, in the absence of a pregnancy (often associated with uterine infection); or initial pregnancy followed by embryo loss which will result in high progesterone, now in the absence of a pregnancy. In all of these cases a higher frequency of measurement (i.e. daily) will help reduce these problems, but do not completely overcome the limitations of the use of P4 as an indicator of pregnancy state.

#### Oestrous synchronisation and ovulation synchronisation

Traditional oestrous synchronisation methods (i.e., prostaglandin only programmes and 12-day progesterone programmes) were designed to synchronise oestrus, but generally still required observation of oestrus to optimize timing of mating and pregnancy rates. As an exception to this two injections of prostaglandin 11 days apart in maiden heifers can work with fixed-time insemination (FTAI) at 72 and 96 h or alternatively at 72 h, and then intensively observe for oestrus for a further 3–4 days and inseminate those late coming into oestrus, in response to standing oestrus (using the am-pm rule) [51]. This protocol in cows required observation for oestrus after the second prostaglandin injection.

Ovulation synchronisation programmes were designed to facilitate use of FTAI in herds without significant investment of time and labour into oestrous detection. These were developed from the early 1990s onwards [45]. They are more appropriate to large non-seasonal herds where calving to calving intervals are somewhat less relevant to economic performance of the herd and often calving intervals are allowed to extend beyond 400–420 days. The major issues for a basic ovulation synchronisation (OVSYNCH) programme is that conception rates to a single round of OVSYNCH are approximately only 30% [57, 58]; and in a European context are relatively expensive. Strategies to improve pregnancy rates have been developed (e.g., Double OVSYNCH and presynchronisation-ovulation synchronisation (PRESYNCH-OVSNCH) that are acceptable in many US herds (46 and 41% conception rates, respectively [59]), but incur substantial costs in terms or time, drug costs, compliance and public perceptions (around routine use of hormones in cattle production) that leave their use questionable in European dairy herds. For seasonal herds the treatment time required for OVSYNCH, PRESYNCH-OVSYNCH and Double OVSYNCH protocols are too long relative to the conception rates that can be achieved. Progesterone based programmes (e.g., 7 or 8 day protocol) using an intravaginal device incorporating GnRH at the start and PGF at the end (Day 7) gives better results in terms of synchronisation and pregnancy rates in healthy cows [60].

#### Pregnancy detection

##### Direct methods of pregnancy detection

Various methods are available to determine pregnancy status, these include return to oestrus [61], rectal palpation of the reproductive tract [62, 63] and ultrasound scanning to observe the reproductive tract [64, 65]. In practice return to oestrus is fraught by the difficulties associated with oestrous observation, so currently most pregnancy detection in cows is carried out by ultrasound scanning of the reproductive tract to detect the presence or absence of the early embryo and foetal fluid. Using this method pregnancy status is generally determined from day 28 onward of pregnancy. This method while routinely used, is too late to allow rebreeding at the optimal time (i.e., 18 to 24 days post initial AI) for non-pregnant cows as the normal oestrous cycle is 18 to 24 days [11].

Ideally an early pregnancy test would:

- Have high sensitivity (ie correctly identify pregnant cows)
- Have high specificity (ie correctly identify non-pregnant cows)
- Be inexpensive to conduct
- Be a simple cow-side test (ie usable in field conditions)
- Determine pregnancy status in a timely manner (ideally at the time of performing the test); (list modified from Fricke et al. [66]).

##### Indirect methods for pregnancy detection in dairy cows

Indirect methods for early pregnancy diagnosis use qualitative or quantitative measures of hormones or conceptus-specific substances in maternal body fluids as indirect indicators of the presence of a viable pregnancy [67, 68]. Commercially available indirect methods for pregnancy diagnosis in dairy cows include milk progesterone tests and tests for pregnancy-associated glycoproteins (PAGs) in blood or milk [67, 68].

Progesterone assays are more useful as a non-pregnancy test on day 21 [67]. However, it is inaccurate as a test for pregnancy as reversion to low P4 in non-pregnant cows is highly variable due to early embryonic losses. It has been tried commercially, but has not survived due to these problems. In-line P4 testing (as mentioned earlier) has potential if the costs of repeated analyses can become competitive.

PAG measurement is a viable method of determination of pregnancy status in dairy cows [68], however, accuracy of PAG detection is only good after day 35 to 40. Interference may also occur from PAG carry over from previous pregnancy for 40–50 days giving rise to a risk of false positives. It also may give false positive results after embryo loss.

**Fig. 3:**
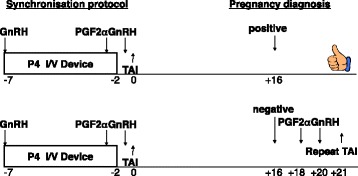
Potential strategy to re-synchronise and re-breed dairy cows after an early pregnancy diagnosis result (day 16; GnRH = gonadotrophin-releasing hormone; PGF2α = prostaglandin F2α; I/V = intravaginal; TAI = fixed-time artificial insemination)

Work described in UK Patent Application No.1520248.4 has led to the development of a test based on glycan diagnostics using the IgG fraction in milk. This technology can detect pregnancy status from as early as day 16 and has led to a priority patent filing (filed 17 November 2015; UK Patent Application No.1520248.4). Importantly early detection of pregnancy status would allow a strategy to resynchronise and rebreed cows by day 21 post the initial unsuccessful insemination (Fig. 3).

### The male side of the story

Although most evidence suggests the pressure on reproductive efficiency in modern dairy herds is primarily related to the high producing females, it is obvious that the role of the male should not be forgotten. While veterinary practitioners often overlook the importance of this side of the coin, farmers often blame issues like sperm quality and the expertise of the AI-technician, mostly because it is human nature to prefer blaming someone else instead of being critical against personal shortcomings. In a study examining the pregnancy outcome of 5883 inseminations, 1 of the 35 bulls that delivered semen was associated with a 2- to 2.5-fold increase in pregnancy rates [69]. In another study from the same group [70] in which the pregnancy results of 10,965 inseminations were scrutinized, not only a difference between bulls but also dramatic differences among inseminators were observed. The likelihood of a pregnancy were almost 4 times lower when a cow was inseminated by the worst compared with the best inseminator.

The success of an insemination depends amongst other factors on the deposition of appropriate numbers of sperm with a good fertilizing capacity at the appropriate site in the reproductive tract at the appropriate time in relation to ovulation. The fertility potential of an artificial insemination dose is a function of the quantity, quality, and health status of the semen contained therein. It is the task of the AI-industry to continue to maintain intense quality control programmes to ensure cryopreserved semen doses released for sale are disease free and meet the above mentioned criteria. A large survey of semen processing practices at AI companies in multiple countries reported that the average cryopreserved AI dose contains approximately 20 × 10^6^ total spermatozoa [71]. This has been estimated to be on average 2 to 20 times greater than estimates of minimum threshold numbers required to guarantee normal fertilisation rates. Remarkably, bulls that are known to produce marginal quality semen often achieve below average fertility despite compensatory increases in number of spermatozoa per dose and vice versa [72].

In 2003 Pace [73] reviewed the technological advances that have been adopted by the AI industry since establishment in the late 1930s and concluded that ‘from a technological standpoint, the dairy industry is receiving the highest quality semen ever produced’. Technological advances in semen processing are reflected in fertilisation rates using cryopreserved semen in the late 1990s early 2000s comparable to those reported for non-frozen semen in the 1950s [72]. Innovative techniques recently tested in the AI industry are cryopreservation techniques that improve post-thaw sperm survival and thereby reduce sensitivity to the optimal timing of the insemination. In this regard, microencapsulation of spermatozoa for sustained time release [74] or techniques designed to reduce the magnitude of cryopreservation-induced capacitation [75] have been tested.

Use of semen from sires with proven high-fertility is probably the most obvious and simple recommendation. However, when one starts to use super fertility bulls in a widespread manner on cows suffering from fertility problems, the question arises in what sense the latter may affect the fertility data reported for these bulls. Use of semen from other breeds in which the fertility decline is not as severe a problem as in the Holstein breed, may also be considered as an alternative to improve herd fertility especially if semen is used of bulls from appropriately progeny-tested sires from breeds with comparable production levels [76]. However we should keep in mind that cross-breeding is not per se a genetic improvement and that genetic selection is still strongly recommended within the breeds used.

Recently reports have been published showing that some sires perform better in timed AI scenarios than others [77]. The latter should stimulate the managers and veterinarians to analyze the fertility data of their herd in relation to the use of specific bulls. On the other hand, it is still unknown whether in this way we stimulate the selection towards animals that are better at coping with specific fixed time protocols instead of stimulating the selection towards enhanced fertility.

A further contributing factor is the site of semen deposition. Although the uterine body is generally accepted to be the proper site for semen deposition, in an experiment using contrast radiography to evaluate the accuracy of professional inseminators, the deposition of semen into the uterine body was successful in only 39% of the attempts, while in 25% of the cases, the semen was deposited in the cervix [78]. While several studies observed improved fertility in response to horn breeding (deep bicornual insemination in which the full insemination dose is divided among both uterine horns or deep unicornual insemination following a preceding ultrasound examination to detect the site of the ovulatory follicle in order to allow ipsilateral insemination), most comparative studies indicated no difference [79]. Recently, a new device that significantly facilitates deep intra-uterine insemination was developed [80], although authors were not able to demonstrate ameliorated pregnancy results using this device versus conventional insemination in the uterine body [81]. Deep intra-uterine insemination was tested for the insemination of low cell number doses of sex sorted sperm. Although flow cytometry/cell sorting has been shown to be a reliable procedure to differentiate X- versus Y-chromosome bearing spermatozoa, the use of sexed semen is often only recommended for nulliparous heifers because of disappointing fertility results in multiparous animals. As a conclusion, DeJarnette et al. [72] reviewing available papers, mentioned that the primary concern is to ensure that semen deposition takes place cranially of the internal cervical os. Remarkable in this context is the paper of López-Gatius and Hunter [82] in which authors report about the successful intrafollicular insemination in repeat breeder cows under heat stress. The latter study however needs confirmation with additional studies.

### Usage and availability of “big data”

#### Common practice in dairy science

Before the “Big Data” era, dairy researchers successfully exploited randomised controlled trial data to explore the complex relationship between production and reproduction in dairy cattle [83, 84]. Multiple observational studies have been designed to identify (metabolic) risk factors that influence this relationship in dairy cattle [85]. Multiple studies have been conducted in this area and successfully published in high valued scientific publications [21, 22, 86]. However, as described by Leblanc [87], temporal associations that have been identified, do not imply causation. Many other aspects of the dairy industry have changed over the last decades confounding the relationship. Randomisation does not exclude confounding. The possibility remains that other variables than the treatment may independently be associated with the intervention and even the outcome. Although well-designed randomised controlled clinical trials remain the gold standard when evaluating experimental treatments, the potential of Big Data in dairy science lies in the combination of traditionally collected data with these new forms of data, both at an animal as well as at a population level. In human medicine, this type of data has been described as real-world evidence [88, 89]. The aforementioned abundance of real-world evidence in animals could potentially help unravel complex relationships such as the often described production-reproduction antagonism in dairy cows. A recent survey from Rutten et al. [90] exactly documents the lack of integrated information and decision-making support tools for current technology in dairy research. No single scientific publication was reported up to 2013 in the areas of metabolism and reproduction utilizing “Big Data”. The survey confirms the scientific methodological challenges observed in Big Data analytics.

Techniques used for analysis and visualisation of traditional dairy data are not adequate for Big Data. The volume, velocity, variety, distributed-ness and incremental nature of such data impose challenges on the traditional methods for data analysis.

#### Herd fertility and data management strategies

Historically, the emphasis in veterinary medicine has focused on the individual cow affected with a clinical disease. However, about 30 years ago, it was recognised that subclinical disease was the major cause of economic losses in dairy herds and veterinarians started to investigate the multifactorial nature of these sub-clinical diseases [91]. This turned out to be effective in improving the overall health status of the herd, and hence profitability. This approach was called herd health management and has been implemented in veterinary education for at least 3 decades. Over the same time period, internet and communication technology has emerged and integrated in herd health management to leverage the understanding of cow records. The generation and use of cow related data has occurred for in excess of 100 years. The first reporting of the recording and the collection of milk production data is from a union of dairy farmers in Denmark in 1895 [92]. In 1906, the first US milk recording association was founded [93]. Since the 1950’s, computers have been used as a management tool in dairy farming [94]. Over subsequent decades, dairy herd management software has evolved quickly and the personal computer has emerged as an important management tool to monitor production, reproduction and health [95]. Technologies to collect and store data have been evolving at a quicker pace compared with the speed at which new insights in dairy science have been discovered. The exponentially increased volume and speed at which data is now created, commonly referred to as Big Data, has brought new challenges for research in dairy science. The way researchers have to leverage the power of Big Data has been at the center of attention ever since the publication trend that started around 2009 [96, 97]. How to address these challenges will be the main scope for future research.

#### Available data for dairy practitioners

1. *Official milk recording organisations* are collecting 4 to 8 weekly milk samples to detect milk components. Novel analytical methods are detecting more metabolites to assess (re-) productive performance in milk. As an example, the entire mid-infra-red (MIR) spectrum of milk has been proposed as a predictor for disease in dairy cows [98]. MIR predictions are now readily available for milk composition traits such as milk fat, protein and fatty acids [98]; under development are additional prediction equations to allow prediction of greenhouse gases and novel performance and health traits [99, 100]. Diagnostic services are routinely analyzing a multitude of parameters in blood, milk and faecal samples from dairy cows. Recently, genomics information has become commercially available for both male and female animals creating a new set of data [101]. These so called secondary off-farm data centres, mainly containing milk recording data, genomic and diagnostics information, have been raised in different countries, each containing a subset of data representing the real world of dairy cows [101–103].
2. On farm, conventional and robotic milking systems are equipped with more and better *sensors* that collect information besides the produced amount of milk. Inline sensors are detecting milk composition, somatic cell counts, temperature and colour [104, 105]. Biosensors are collecting novel biomarkers such as progesterone (reproduction), L-lactate dehydrogenase (udder health), urea and beta-hydroxy-butyrate (metabolic health) [106, 107]. Weighing scales and 3 dimensional cameras [108, 109] are capturing the animal’s body weight and body condition score while milking [110, 111]. Ever since the beginning of sensor technology, cows have been equipped with pedometers and accelerometers that capture the animal’s movements in order to predict specific behaviour such as oestrus and disease in dairy cows [90, 112, 113]. Examples of early-stage innovations being applied to dairy cows are ruminal temperature and pH boluses [114, 115], intravaginal temperature sensors [116, 117] and heart rate measurements [118]. The volume or format of the data no longer presents a major constraint, hence the total volume of cow related data that is collected per day has increased rapidly [103, 119, 120].

#### New data sources in the dairy industry

1. The importance of environmental factors such as temperature and humidity in dairy reproduction are undeniable [121–124]. Location based data has become publically available over recent decades, creating the ability to layer physical maps and location-based insights on top of other available data. The approach of combining real-time Internet-of-Things (IoT) devices with historical data analysis are unexploited in dairy science. Thus data streamed from automated data loggers for environmental factors offers new applications in terms of big data collection and use to alter decision making and management [125, 126].
2. The speed and capacity of computer hardware has increased, while costs have decreased [119, 127]. This has led towards easier data recording through cheap mobile devices and high-availability cloud based data-centres that allows a more consistent and accurate capturing of manually entered reproduction, disease and treatment events at cow level. Using this data to build predictive models for anticipating disease outcomes from current treatment plans and refine those models in real time will improve scientific knowledge around treatment efficacy which is limited to observational studies at the moment [128].
3. Furthermore, mobile captured anecdotal and unstructured data from farmers, veterinarians’ notes and other sources is a giant frontier of untapped insights. Nowadays, it has been recognised by researchers that 85% of the world’s information is unstructured, comprised of free-form text, audio and video, rather than neatly organised recognisable fields [129]. Although the need for a standardised disease data input has already been recognized for a long time [130], effective implementation in current software is lacking [128]. Natural language processing consists of multiple computational techniques to process language human-like from machine-readable unstructured texts. This has been successfully applied in human medicine [131, 132], but not yet in dairy science to our knowledge. Capturing and exploiting this data will enrich analysis and insights immensely.

## Conclusions

In conclusion, genetic trends for fertility are improving in dairy cow populations. Numerous future developments are likely over the next 5 to 10 years. These include: i) development of new and novel phenotypes that may be measurable in milk; ii) specific genomic markers; iii) early pregnancy detection; iv) increased use of activity monitors; v) improved breeding protocols; vi) automated inline sensors for relevant phenotypes that become more affordable for farmers; and vii) capturing and mining multiple sources of “big data” available to dairy farmers. These new developments should facilitate improved performance and health of dairy cows in the future.

## Abbreviations

AC: Activity cluster
AI: Artificial insemination
CL: Corpus luteum
EU: European Union
FTAI: Fixed-time artificial insemination
GH: Growth hormone
GnRH: Gonadotrophin-releasing hormone
GplusE: Genotype plus Environment
IGF-I: Insulin-like growth factor I
IgG: Immune-gamma globulin
IoT: Internet of things
IVF: Invitro-fertilisation
MIR: Mid-infra red spectra
NEB: Negative energy balance
OVSYNCH: Ovulation synchronisation
P4: Progesterone
PAGs: Pregnancy-associated glycoproteins
PGF: Prostaglandin-F2alpha
PRESYNCH-OVSYNCH: Presynchronisation-ovulation synchronisation
SNPs: Single-nucleotide polymorphisms
